# Regretting Ever Starting to Smoke: Results from a 2014 National Survey

**DOI:** 10.3390/ijerph14040390

**Published:** 2017-04-06

**Authors:** Pratibha Nayak, Terry F. Pechacek, Paul Slovic, Michael P. Eriksen

**Affiliations:** 1 Georgia State University’s Tobacco Center of Regulatory Science, Atlanta, GA 30308, USA; tpechacek@gsu.edu (T.F.P.); meriksen@gsu.edu (M.P.E.); 2 Division of Health Management & Policy, Georgia State University, Atlanta, GA 30303, USA; 3 Department of Psychology, University of Oregon, Eugene, OR 97403, USA; pslovic@uoregon.edu

**Keywords:** tobacco use disorder, addiction, nicotine, adults, guilt, regret

## Abstract

*Background*: The majority of smokers regret ever starting to smoke, yet the vast majority continue to smoke despite the fact that smoking kills nearly 50% of lifetime users. This study examined the relationships between regret and smoker characteristics, quit history, risk perceptions, experiential thinking, and beliefs and intentions at time of smoking initiation. *Methods*: Data from the 2014 Tobacco Products and Risk Perceptions Survey, a nationally representative survey of United States adults, were analyzed to provide the latest prevalence estimates of regret and potential predictors. Relationships among predictor variables and regret were analyzed using correlations, *t*-tests, and multinomial logistic regression. *Results*: The majority of smokers (71.5%) regretted starting to smoke. Being older and non-Hispanic white were significant predictors of regret. Smokers having a high intention to quit, having made quit attempts in the past year, worrying about getting lung cancer, believing smoking every day can be risky for your health, perceiving a risk of being diagnosed with lung cancer during one’s lifetime, and considering themselves addicted to cigarettes were significant predictors of regret for smoking initiation. *Conclusions*: This study provides updated prevalence data on regret using a national sample, and confirms that regret is associated with perceived risk. The findings from this study can be used to inform smoking intervention programs and support the inclusion of smoker regret in cost–benefit analyses of the economic impact of tobacco regulations.

## 1. Introduction

Smoking is a significant health burden [1,2] and causes more than twice as many deaths annually as human immunodeficiency virus and AIDS, alcohol abuse, car accidents, use of illicit drugs, and suicide combined [3]. Chronic smoking can reduce life expectancy by more than 10 years, although smokers who quit before the age of 45 years can regain this potentially lost decade in life expectancy [4]. Despite these facts, the majority of smokers continue to smoke and remain at increased risk for adverse health outcomes.

Smokers often regret ever picking up this potentially deadly habit [5,6,7], and most smokers desire to quit [8]. Quit attempts are common, with almost half of smokers indicating they made a quit attempt in the past year [9], although only 6% of those who try to quit succeed [9,10]. Festinger’s theory [11] on cognitive dissonance provides a framework for understanding the discrepancy between one’s belief in desiring to quit while continuing to smoke [12]. According to this theory, individuals try to seek consistency with their beliefs. In situations where their behaviors are inconsistent with their beliefs, they experience discomfort that motivates them to engage in altering their beliefs to reduce this dissonance. Previous studies have examined how smokers reconcile this discrepancy and its relationship with the negative feeling of regret from starting to smoke [5,7,13,14].

Studies indicate that smokers tend to experience cognitive dissonance and engage in dissonance reduction activity to help justify their smoking [15,16]. Smokers justify their behavior by either elevating positive aspects of the dissonant behavior (e.g., smoking reduces stress, smoking is enjoyable) or minimizing thoughts of undesirable negative consequences (e.g., “even non-smokers die of lung cancer”, “Smoking won’t kill me”) [13,15,17,18,19]. A previous cross-sectional study suggests that perceptions of health risks are specific to smoking behavior, such as the decision to initiate smoking, continue to smoke, or quit smoking [20]. Higher perceived risk of smoking-related conditions and harms from smoking plays important role in quitting behavior [21]. Risk perception plays a stronger role in influencing quitting versus initiating smoking [22].

The work of both Weinstein and Slovic demonstrates that smokers may misunderstand smoking risks [14,22,23,24,25,26,27,28]. Weinstein states that “smoking cannot be interpreted as a choice made in the presence of full information about the potential harm” [27]. Their studies find that smokers underestimate their personal risk and often misperceive the nature and severity of tobacco-related diseases [27]. Smokers often do not think carefully about risks from smoking when they first begin to smoke [28,29,30]. Youth tend to initiate smoking based on the notion that it is a fun and exciting behavior; they often learn of the health risks from exposure after initiation through exposure to anti-smoking media messages about the hazards of smoking [29]. Youth often repudiate their earlier decision of smoking and feel guilty for having ever started smoking [5]. Regret is experienced as a negative cognitive-based emotion that arises when comparing one’s addiction to smoking and the feeling of an alternative reality where s/he had never started smoking [31]. Regret about smoking differs by characteristic, such as having a stronger intention to not smoke, being a long-term and heavy smoker, believing that an addiction to cigarettes is occurring, perceiving a higher risk for harm, and higher number of attempts to quit smoking [32].

Studies of smoker regret were published by Slovic in 2001 [14], Fong et al. in 2002 [5], and O’Connor et al. in 2010 [33]. Slovic, Fong and O’Connor examined a U.S. sample while the other two studies [7,13] focused on Asian countries. The present study builds upon this prior work and had three main goals: to provide the latest estimates of regret experience among U.S. adults, to identify factors that predict the experience of regret, and to examine the relationship of regret with quit intention, quit attempts, risk perception including relative harm perception, experiential thinking in the decision to smoke and addiction perception, and beliefs during smoking initiation.

## 2. Materials and Methods

### 2.1. Survey Sample

This study was approved by Georgia State University’s (GSU) Institutional Review Board (45 CFR 46). The study used data from the 2014 Tobacco Products and Risk Perceptions Survey (TPRPS) commissioned by GSU’s Tobacco Center of Regulatory Science and administered by GfK. A probability sample was drawn from GfK’s KnowledgePanel and was weighted to represent non-institutionalized U.S. adults, about 97% of the U.S. adult population [34].

The final stage completion rate was 74.4%, yielding a total of 5717 respondents who completed the survey. In order to adjust for sampling and non-sampling error, data were weighted using an iterative proportional fitting (raking) procedure. Demographic and geographic distributions from the most recent Current Population Survey were employed as benchmarks for adjustment and included age, sex, education, annual household income, race/ethnicity, census regions, and metropolitan area. Reconfirmed smokers were then weighted to represent smokers ages 18+ for the same demographics used for the general population respondents. The present study was restricted to only current smokers (*n* = 1349).

### 2.2. Measures of Smoking

Current smokers were respondents who reported lifetime smoking levels of at least 100 cigarettes and smoking “every day” or “some days”. Two items were used to define level of smoking: “Do you currently smoke cigarettes every day or some days?” and “On days you smoke, how many cigarettes do you smoke on average?” Level of smoking was defined as “non-daily—very light”, “non-daily—light”, “daily—very light”, “daily—average”, “daily—heavy”, and “daily—very heavy”. We used the following cutoff’s to define the smoking level categories; reported smoking some day and 1–4 cigarettes; some day and 5 or more cigarettes ; daily and 1–9 cigarettes ; daily and 10 to 14; daily and 15 to 24; and daily and 25 or more cigarettes , respectively.

#### 2.2.1. Regret

Smoker regret was measured using the variable “If you had it to do over again, would you start smoking cigarettes?” with response options of “yes”, “no”, and “don’t know”. The “yes” responses were defined as “not having regret”, “no” responses were defined as “having regret”, and “don’t know” responses remained the same.

#### 2.2.2. Intention to Quit

Intention to quit cigarette smoking was measured using responses from a six-point scale ranging from “plans to quit within a week” to “not at all”. Responses were categorized into dichotomous responses of “high intention to quit”, (responses: “within a week”, “<1 month”, “within 6 months”, and “quitting in less than a year”), and low intention to quit (responses: “will plan to quit someday” or “not planning to quit”). Respondents were also asked if they had attempted to quit smoking in the past year (responses: “yes” or “no”).

#### 2.2.3. Perception of Harm from Smoking

Harm perceptions from smoking were assessed using three items. The first item, “How often do you worry about getting lung cancer?” was measured with responses on a four-point scale (“rarely or never”, “sometimes”, “often”, or “all of the time”). Opinions about the risks of smoking every day and once in a while were collected using two separate items and responses were measured on a four-point scale (not at all risky, a little risky, somewhat risky, or very risky). A mean score was independently calculated for each of three items across regret status.

Relative harm perceptions were assessed by asking respondent agreement with the question “How would smoking impact someone who starts to smoke regularly at age 16?” using two items: “There is usually no risk to this person at all for the first few years” and “If someone wants to smoke, they should be able to because it is a personal choice”. Responses were measured on a five-point scale: “strongly disagree”, “somewhat disagree”, “neither agree nor disagree”, “somewhat agree”, and “strongly agree”. Respondents were also provided with the option “Don’t know”, which was excluded from the analyses.

We also assessed optimism bias for lung cancer using the question item “Compared to others your age who currently smoke cigarettes, what do you think are your chances of being diagnosed with lung cancer during your lifetime?” Responses were collected on a five-point response scale (“at much less risk”, “at less risk”, “at the same risk”, “at higher risk”, and “at much higher risk”).

#### 2.2.4. Experiential Thinking in Decision to Smoke and Addiction Perception

To assess decision-making related to smoking, respondents were asked “How much do you think about the health effects of smoking cigarettes now?”, which was measured using the response options “not at all”, “a little”, and “a lot”. Addiction perceptions were measured using the item “Do you consider yourself addicted to cigarettes?” with response options of “not at all”, “yes, somewhat addicted”, and “yes, very addicted”.

#### 2.2.5. Beliefs and Intentions during Smoking Initiation

This construct was measured using the item “Thinking again about the first time you ever smoked a cigarette, did your smoking happen…” and response options “with much thought”, “without much thought”, or “I don’t know”. Respondents were also asked “When you first started smoking cigarettes, did you think more about…”, with response options, “yes” and “no” for “how smoking would affect your future health” and “about how you were trying something new and exciting”. Intention to continue smoking was measured using the item “When you first started smoking cigarettes, how long did you think you would continue to smoke?”; responses included “less than a year”, “1 year or more”, “I didn’t think about it”, or “I don’t know”.

#### 2.2.6. Demographics

The respondents’ demographic characteristics were obtained from profile surveys administered by GfK to all KnowledgePanel panelists. Demographic characteristics included self-reported sex, age, race/ethnicity, educational attainment, annual household income, and health status. Information on tobacco use, intention to quit, and attempts to stop smoking were collected through self-reported Tobacco Product Risk Perception Survey.

### 2.3. Data Analysis

All analyses were conducted using SAS survey procedures in SAS^®^ software version 9.3 (SAS Institute Inc., Cary, NC, USA) to account for complex survey design. All analyses were weighted to obtain population estimates. To examine differences in proportions across demographic variables, chi-square tests were conducted across the dependent variable “regret status” and 95% confidence intervals for proportions were estimated using the logit method. *T*-tests examined differences in means across perception of harm from smoking and relative harm perceptions. Multinominal logistic regression models controlling for demographic information were conducted to examine associations between regret status and level of smoking, intention to quit, quit attempts, perception of harm from smoking, relative harm perception, experiential thinking in the decision to smoke, and addiction perception.

## 3. Results

The study sample consisted of 1331 current smokers who reported on the regret statement “If you had to do over again, would you start smoking cigarettes?” Table 1 presents respondent characteristics by regret statement response; a vast majority of respondents indicated having regret for having ever started smoking (71.5%; 95% CI: 68.6%–74.4%). Approximately three-fourths of current smokers reported smoking daily. Overall, 13.4% (95% CI: 11.4%–15.6%) indicated having no desire to quit and 61.8% (95% CI: 58.7%–64.8%) reported not having made any quit attempts in the past year. Sex, age, race/ethnicity, level of smoking, intention to quit, and having made a quit attempt in the last year were significant predictors of regret.

### 3.1. Perception of Harm from Smoking

**Figure 1:**
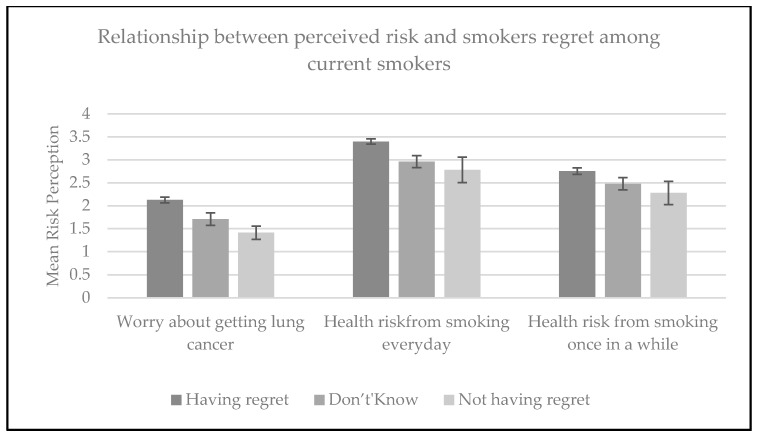
Smokers’ regret and their perception of harm from smoking. Higher means indicate higher perceived risk.

Three of the risk perception variables were very strong predictors of regret (*p* < 0.001): worry about getting lung cancer, the perception that there is an increased health risk from smoking every day, and the perception that there is an increased health risk from smoking once in a while. For all three predictors, higher perceived risk was correlated with a higher likelihood of experiencing regret (Figure 1).

Adjusting for demographic characteristics, worry that smoking would cause lung cancer was a strong predictor of regret (aOR: 5.3; 95% CI: 3.4–8.3) (Table 2). Respondents who perceived that there is an increased health risk from smoking every day (aOR: 2.6; 95% CI: 1.9–3.4), as well as once in a while (aOR: 1.9; 95% CI: 1.4–2.6) (Table 2) were more likely to experience regret.

#### Relative Harm Perception

**Figure 2:**
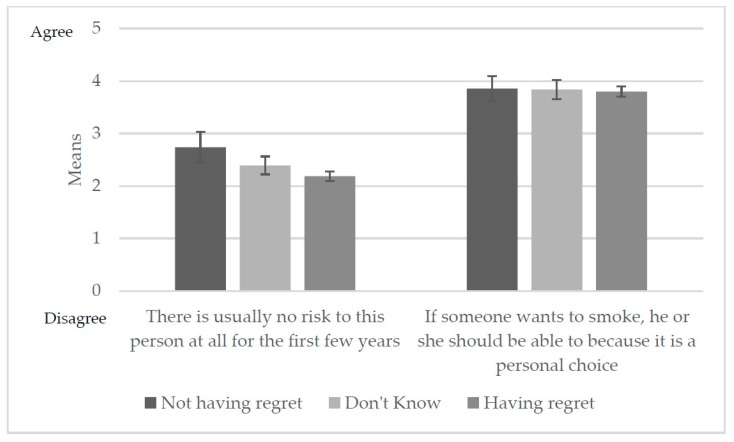
Relative harm perception by smoker’s regret experience.

Respondents who reported regret had lower means on the item “There is no risk to the person at all for the first few years of smoking” (Figure 2). Multivariable regression analysis indicated this relationship was a significant predictor of having regret; respondents who agreed that there is no risk to the person who smokes regularly were less likely to have regret (aOR: 0.7; 95% CI: 0.6–0.8) (Table 2). The belief that smoking should be a personal choice was not a significant predictor of regret.

Those with regret also had higher means for perceived risk of being diagnosed with lung cancer in their lifetime compared to those who did not have regret for smoking (mean (SE): 3.2 (0.04) vs. 2.7 (0.15), respectively). Respondents experiencing regret and those who did not know their regret status were more likely to perceive a higher risk for being diagnosed with lung cancer in their lifetime than those who did not regret smoking (aOR: 1.6; 95% CI: 1.3–2.2) (Table 2).

### 3.2. Experiential Thinking in the Decision to Smoke and Addiction Perceptions

Overall, 39.3% of current smokers in our study sample reported that they thought a lot about how smoking could harm their health in the present day (Table 3). This association varied by regret status, with 48.3% of those having regret and 14.9% of those not having regret responding that they thought a lot about how smoking could harm their health (Table 3).

Worrying a lot about the effect of smoking cigarettes on their health was a strong predictor of regret, at both bivariate and multivariate levels (having regret vs. not, aOR: 8.0, 95% CI: 5.2–12.3; don’t know vs. not having regret, aOR: 2.1, 95% CI: 1.3–3.2) (Table 2). About 44% of included respondents believed they were very addicted to cigarettes; a higher proportion of these respondents reported having regret versus not having regret (49.7% vs. 24.5%, respectively). Individuals who considered themselves addicted to cigarettes compared to those who did not consider themselves addicted were more likely to report experiencing regret (aOR: 3.5; 95% CI: 2.4–5.2).

### 3.3. Beliefs and Intentions during Smoking Initiation

Most respondents initiated smoking without thinking much about their behavior or future health. Some respondents felt smoking was exciting and new, but most did not think much about health or excitement when they started smoking (Table 4). More than half of the respondents indicated they did not think about how long they would continue to smoke. Adjusting for all demographic variables, those who initiated smoking without much thought were more likely to experience regret (aOR: 2.8, 95% CI: 1.7–4.7) (Table 5) compared to those who initiated with much thought.

### 3.4. Intention to Quit and Regret

As presented in Table 5, low (versus high) intention to quit was inversely associated with having regret (aOR: 0.2, 95% CI: 0.1–0.3). In addition, those who had made any quit attempts in the past year had higher odds of regret for smoking compared to those who had made no quit attempts (aOR: 2.9; 95% CI: 1.8–4.8).

## 4. Discussion

This study provides estimates from a recent national survey of US adults on the regret smokers experience from continuing to smoke and predictors associated with this regret. The findings indicate that the vast majority of smokers regret having started smoking [5,13,33]. Perceiving health risks from smoking was associated with having regret. Smokers with higher levels of regret were more likely to perceive themselves at higher risk for being diagnosed with lung cancer during their lifetime compared to those not experiencing regret. Regretful smokers reported spending more time thinking about the impact of smoking on their health and were more likely to consider themselves addicted to cigarettes. In addition, regretful smokers reported that their smoking initiation happened without much thought, including a lack of thought about how long they would continue to smoke when they first started smoking cigarettes.

Consistent with previous research [5,13,33], the prevalence of regret was high among smokers in this study; across studies, reported regret ranges from 79% to 92% (Malaysia (79%), the United Kingdom (89%), Australia (90%), Thailand (92%), and Canada (92%)). Fong et al. reported that, among United States smokers, the prevalence of smoker regret was 91% in 2004, whereas a study by O’Connor using 2010 U.S. data reported 85% regret among a similar sample of U.S. adults. Our study estimated that 71% of smokers reported experiencing regret, and 21% reported not knowing their regret status. Only about 8% said they experienced no regret. Our findings may differ somewhat from the previous studies due to the use of a 3-point (vs. 5-point) scale to collect regret status. Moreover, the data in Fong et al. were collected using a telephone survey while data from this study were collected from a web panel; therefore, survey mode effects could have contributed to variations in reported estimates.

Regret was experienced at a higher rate among smokers who perceived a higher risk of suffering from lung cancer and other health risks. These findings indicate that people who perceived a higher relative risk and risk difference of lung cancer from smoking were more likely to regret having smoked. The overall perceived risk for occasional smoking was lower than for daily smoking. Young people perceive the risk of occasional smoking as “relatively risk-free” [28] (p. 220) and relatively low risk when compared to other substance use, such as alcohol and drug use. Optimism about avoiding the cumulative risk of and avoiding harm from occasional smoking encourages experimentation and subsequent progression to daily or heavier smoking [28]. In this study, smokers did report that their habit may expose them to increased future risk for lung cancer, and those with higher perceived risk were more likely to experience regret. These findings were similar to Fong et al.’s [5] findings that worrying about smoking damaging future health was a predictor of regret and believing that smoking lowers their quality of life. Although individuals in the current study were more likely to believe in the benefits of quitting and make quit attempts, many reported they were potentially addicted to smoking. Concern with how smoking affects health increases fear of future consequences from smoking and regret.

One limitation of the study design is that it does not assess the causal direction of associations between regret and risk behavior. A second limitation pertains to recall bias; respondents were asked to recall their early life experience pertaining to initial smoking behavior and their beliefs and intentions during initiation. Although people usually remember their initial experience with cigarettes [35,36], the time lapse between smoking initiation and survey response may influence recall on risk perception items. A third possible limitation of these data comes from its sampling frame, which uses a web panel to draw its sample. The use of the web panel may raise concerns about sample representativeness, especially if the panel has been used in prior tobacco research, which potentially affects the generalizability of the study findings.

## 5. Conclusions

High levels of regret about smoking behaviors characterize the struggle of smokers who desire to quit smoking. Smokers may not fully understand the consequences of their actions, nor perceive their future health risks when they begin smoking. A lack of understanding among youth concerning the risks that smoking entails supports the use of graphic warning labels on tobacco products. These results suggest that these labels be based on an understanding of smoker psychology and use affective imagery to elicit negative feelings toward smoking. All individuals, young and old, including non-smokers, need to be made aware of the overwhelming regret and dissatisfaction that smokers experience in association with their decision to smoke. It is important for smoking intervention programs to communicate not only the health risks associated with smoking, but also the addiction risk, and the psychological distress that results from not being able to control a behavior that one regrets initiating. The findings show that smokers do not find smoking pleasurable and derive little benefit from continuing to smoke rather than experience “lost pleasure” from quitting smoking. The present findings support the inclusion of smoker regret in cost-benefit analyses of tobacco control regulatory actions [6].

## Figures and Tables

**Table 1:** Smoker’s regret by respondent characteristics among U.S. adults, 2014.

Respondent Characteristics	Overall	Smoker Self-Reported Regret Status (*N* = 1331)		
	*n* = 1331 % (95% CI)	Not Having Regret *n* = 99; % (95% CI)	Don’t Know *n* = 247; % (95% CI)	Having Regret ** *n* = 985; % (95% CI)
Total	-	7.8 (6.10–9.50)	20.7 (18.0–23.3)	71.5 (68.6–74.4)
Sex *				
Male	51.5 (48.4–54.7)	63.5 (52.4–73.4)	54.7 (47.4–61.8)	49.3 (45.7–53.0)
Female	48.5 (45.3–51.6)	36.5 (26.6–47.7)	45.3 (38.2–52.6)	50.7 (47.0–54.4)
Age (years) *				
18–34 years	31.1 (28.0–34.2)	30.3 (20.8–41.9)	38.2 (31.1–45.8)	29.1 (25.6–32.8)
35–54 years	41.1 (38.1–44.3)	51.3 (40.0–62.4)	37.1 (30.4–44.3)	41.2 (37.6–44.9)
≥55 years	27.8 (25.3–30.5)	18.4 (12.0–27.3)	24.8 (19.5–30.9)	29.7 (26.7–32.9)
Race/ethnicity *				
White, Non-Hispanic	61.1 (57.8–64.3)	47.5 (36.6–58.7)	56.3 (48.9–63.5)	64.0 (60.2–67.6)
Other	38.9 (35.8–42.2)	52.5 (41.3–63.5)	43.7 (36.5–51.1)	36.0 (32.4–39.8)
Education				
High school or less	57.6 (54.5–60.6)	54.5 (43.2–65.2)	63.3 (56.4–69.8)	56.2 (52.6–59.8)
Some college	32.3 (29.5–35.2)	31.6 (22.7–42.2)	28.0 (22.2–34.6)	33.6 (30.3–37.0)
College degree **^+^**	10.2 (8.6–12.0)	13.9 (8.00–23.3)	8.7 (5.80–12.9)	10.2 (8.40–12.3)
Annual household income				
<$30,000	42.5 (39.4–45.7)	45.4 (34.3–56.9)	40.7 (33.7–48.1)	42.8 (39.1–46.5)
$30,000–$60,000	30.0 (27.3–32.9)	28.3 (19.6–39.1)	33.9 (27.4–41.0)	29.1 (26.0–32.4)
>$60,000	27.5 (24.8–30.2)	26.3 (17.9–37.0)	25.5 (19.8–32.1)	28.1 (25.1–31.4)
Perceived health status				
Excellent/Very good	32.3 (29.3–35.4)	38.2 (27.3–50.4)	33.0 (26.4–40.4)	31.4 (28.1–35.0)
Good	44.6 (41.4–47.8)	35.9 (26.0–47.1)	46.5 (39.4–53.8)	44.9 (41.2–48.7)
Fair/Poor	23.2 (20.5–26.0)	26.0 (17.4–36.9)	20.5 (15.1–27.2)	23.7 (20.6–27.0)
Level of smoking *				
Non-Daily—Very light	13.7 (11.6–16.1)	20.7 (13.2–31.1)	19.1 (13.7–26.0)	11.4 (9.20–14.0)
Non-Daily—Light	9.1 (7.5–11.1)	5.1 (2.10–11.9)	9.2 (5.80–14.3)	9.6 (7.60–12.0)
Daily—Very light	19.4 (16.9–22.1)	27.8 (18.6–39.3)	20.4 (15.0–27.1)	18.1 (15.4–21.3)
Daily—Average	22.7 (20.1–25.5)	19.6 (12.2–30.0)	25.0 (19.2–31.9)	22.3 (19.4–25.6)
Daily—Heavy	27.3 (24.6–30.1)	20.8 (12.8–31.9)	19.6 (14.6–25.9)	30.2 (27.0–33.6)
Daily—Very heavy	7.8 (6.40–9.60)	6.1 (2.40–14.7)	6.6 (4.40–10.0)	8.4 (6.60–10.6)
Intention to quit *				
Quit in <1 month	9.5 (7.80–11.4)	5.6 (2.20–13.6)	5.8 (3.20–10.3)	11.0 (8.9–13.4)
Quit in 6 months to 1 year	34.6 (31.7–37.6)	12.2 (6.70–21.3)	19.9 (14.6–26.5)	41.3 (37.8–45.0)
Quit someday	42.6 (39.4–45.8)	40.0 (29.4–51.7)	52.2 (44.9–59.3)	40.1 (36.4–43.8)
Never quit	13.4 (11.4–15.6)	42.2 (31.5–53.7)	22.2 (17.0–28.3)	7.7 (6.00–9.70)
Attempted quitting in the past year *				
Yes	38.2 (35.2–41.3)	24.2 (15.9–35.1)	21.2 (15.7–27.9)	44.7 (41.0–48.3)
No	61.8 (58.7–64.8)	75.8 (64.9–84.2)	78.8 (72.1–84.3)	55.3 (51.7–59.0)

Notes: * *p* < 0.05, indicates significance between groups; ** Having regret was defined as smokers who reported “no” on the statement “If you had to do it all over again, would you start smoking cigarettes?”; **^+^** Bachelor’s degree or higher.

**Table 2:** Multinomial logistic regression analysis assessing predictors of regret.

	** Having Regret vs. Not Having Regret aOR (95% CI)	Don’t Know vs. Not Having Regret aOR (95% CI)
Perception of harm from smoking		
Worry about getting lung cancer	**5.3 (3.4–8.30)**	**2.4 (1.5–3.80)**
Smoking every day can be risky for your health	**2.6 (1.9–3.40)**	1.3 (1.0–1.80)
Smoking only once in a while can be risky for your health	**1.9 (1.4–2.60)**	1.3 (0.9–1.80)
Relative harm perception		
There is usually no risk at all for the first few years	**0.7 (0.6–0.80)**	**0.8 (0.7–0.98)**
If someone wants to smoke, he or she should be able to because it is a personal choice	0.9 (0.8–1.10)	1.0 (0.8–1.20)
Compared to others your age who currently smoke cigarettes, what do you think are your chances of being diagnosed with lung cancer during your lifetime?	**1.6 (1.3–2.20)**	1.2 (0.9–1.60)
Experiential thinking in decision to smoke and addiction perception		
How much do you think about the health effects of smoking cigarettes now?	**8.0 (5.2–12.3)**	**2.1 (1.3–3.20)**
Do you consider yourself addicted to cigarettes?	**3.5 (2.4–5.20)**	**1.5 (1.03–2.3)**

Notes: Bold type means the variable was a significant predictor of regret, after controlling for age, sex, education, and ethnicity/race. ** Having regret was defined as smokers who reported “no” on the statement “If you had to do it all over again, would you start smoking cigarettes?” In addition, please note that multinomial logistic regression analyses controlling for demographic information were conducted individually for each survey item listed in the table.

**Table 3:** Experiential thinking in decision to smoke and addiction perceptions by regret status.

	Not Having Regret	Don’t Know	** Having Regret
	% (95% CI)	% (95% CI)	% (95% CI)
How much do you think about the health effects of smoking cigarettes now? * (*n* = 1279)			
Not at all	41.9 (31.0–53.6)	20.0 (14.8–26.6)	5.13 (3.70–7.10)
A little	43.3 (32.6–54.6)	65.0 (57.3–71.9)	46.56 (42.9–50.3)
A lot	14.9 (8.20–25.5)	15.0 (10.0–21.8)	48.32 (44.6–52.0)
Do you consider yourself addicted to cigarettes? * (*n* = 1286)			
Not at all	30.4 (21.2–41.6)	21.2 (15.5–28.3)	5.51 (4.00–7.60)
Yes, somewhat addicted	45.1 (34.2–56.6)	48.7 (41.0–56.4)	44.82 (41.2–48.5)
Yes, very addicted	24.5 (15.2–36.9)	30.1 (23.5–37.7)	49.68 (46.0–53.4)

Notes: * *p* < 0.0001; ** Having regret was defined as smokers who reported “no” to the statement “If you had to do it all over again, would you start smoking cigarettes?”.

**Table 4:** Smoker’s beliefs and intentions during smoking initiation by regret status.

	Not Having Regret	Don’t Know	** Having Regret
	*n*, % (95% CI)	*n*, % (95% CI)	*n*, % (95% CI)
Thinking again about the first time you ever smoked a cigarette, did your smoking happen with much thought or without much thought? * (*n* = 1324)			
With much thought	26.6 (18.0–37.3)	12.8 (8.70–18.4)	10.6 (8.70–12.9)
Without much thought	68.3 (57.3–77.6)	62.2 (54.8–69.0)	81.2 (78.1–83.9)
I don‘t know	5.2 (2.20–11.7)	25.1 (19.1–32.2)	8.2 (6.30–10.7)
When you first started smoking cigarettes, did you think more about how smoking would affect your future health or about how you were trying something new and exciting? * (*n* = 1325)			
Thought more about future health	8.5 (3.88–18.2)	5.5 (2.50–11.6)	3.9 (2.60–6.00)
Thought more about trying something new and exciting	26.7 (17.6–38.3)	35.3 (28.6–42.6)	43.0 (39.4–46.6)
I did not think about either of these	64.7 (52.9–75.0)	59.2 (51.8–66.3)	53.1 (49.4–56.8)
When you first started smoking cigarettes, how long did you think you would continue to smoke? * (*n* = 1325)			
Less than a year	11.6 (6.10–20.9)	12.3 (7.90–18.6)	22.5 (19.4–25.9)
1 year or more	17.0 (10.3–26.7)	7.1 (4.10–12.0)	7.7 (5.90–9.90)
I didn‘t think about it	62.4 (51.1–72.5)	56.1 (48.7–63.2)	58.2 (54.4–61.8)
I don‘t know	9.0 (4.40–17.6)	24.5 (18.7–31.5)	11.7 (9.60–14.2)

Notes: * *p* < 0.05; ** Having regret was defined as smokers who reported “no” on the statement “If you had to do it all over again, would you start smoking cigarettes?”.

**Table 5:** Multinomial logistic regression analysis of regret.

	** Having Regret vs. Not Having Regret aOR (95% CI)	Don’t Know vs. Not Having Regret aOR (95% CI)
Thinking again about the first time you ever smoked a cigarette, did your smoking happen with much thought or without much thought?		
With much thought	ref	ref
Without much thought	**2.8 (1.7–4.7)**	**2.1 (1.2–3.9)**
I don’t know	**2.8 (1.1–7.3)**	**8.1 (3.0–21.9)**
When you first started smoking cigarettes, did you think more about how smoking would affect your future health or about how you were trying something new and exciting?		
Thought more about future health	ref	ref
Thought more about trying something new and exciting	**2.8 (1.04–7.7)**	2.2 (0.7–7.3)
I did not think about either of these	1.4 (0.65–3.8)	1.9 (0.7–5.8)
When you first started smoking cigarettes, how long did you think you would continue to smoke?		
Less than a year	ref	ref
1 year or more	**0.2 (0.1–0.5)**	0.4 (0.1–1.0)
I didn’t think about it	**0.5 (0.3–0.99)**	1.2 (0.5–2.6)
I don’t know	0.7 (0.3–1.7)	2.8 (1.0–7.8)
Level of smoking		
Non-Daily—Very light	ref	ref
Non-Daily—Light	2.6 (1.0–6.9)	1.8 (0.6–5.2)
Daily—Very light	1.1 (0.5–2.2)	0.9 (0.4–1.9)
Daily—Average	1.6 (0.8–3.3)	1.2 (0.6–2.6)
Daily—Heavy	2.1 (1.1–4.2)	1.1 (0.5–2.2)
Daily—Very heavy	2.5 (0.9–7.3)	1.8 (0.6–5.7)
Intention to quit		
Low vs. high	**0.2 (0.1–0.3)**	0.6 (0.3–1.1)
Quit attempts		
Yes vs. no	**2.9 (1.8–4.8)**	0.9 (0.5–1.7)

Notes: Bold type means the variable was a significant predictor, after controlling for age, sex, education, and ethnicity/race; ** Having regret was defined as smokers who reported ”no” on the statement “If you had to do it all over again, would you start smoking cigarettes?” In addition, please note that multinomial logistic regression analyses controlling for demographic information were conducted for individually for each survey item listed in the table.
